# (4-Chloro­benzohydrazidato-κ^2^ *N*′,*O*)[2-(4-chloro­benzoyl­hydrazono-κ^2^ *N*,*O*)propionato(2−)-κ*O*]oxidovanadium(V)

**DOI:** 10.1107/S1600536809019928

**Published:** 2009-06-06

**Authors:** Hon Wee Wong, Kong Mun Lo, Seik Weng Ng

**Affiliations:** a Department of Chemistry, University of Malaya, 50603 Kuala Lumpur, Malaysia

## Abstract

In the crystal structure of the title compound, [VO(C_7_H_6_ClN_2_O)(C_10_H_7_ClN_2_O_3_)], the V^V^ atom is *N*,*O*-chelated by the chloro­benzoyl­hydrazidate anion and *O*,*N*,*O*′-chelated by the (chloro­benzoyl­hydrazono)propionate dianion. The distorted octa­hedral *trans*-N_2_O_4_ coordination geometry is completed by the vanadyl O atom. In the crystal, mol­ecules are linked by N—H⋯O hydrogen bonds into a linear chain parallel to [010].

## Related literature

For the analogous vanadyl complex without the chlorine substituent in the two ligands, see: Wong *et al.* (2009 ▶).
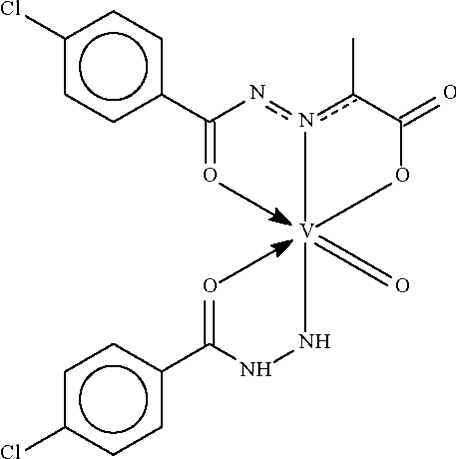


## Experimental

### 

#### Crystal data

[V(C_7_H_6_ClN_2_O)(C_10_H_7_ClN_2_O_3_)O]
*M* *_r_* = 475.15
Monoclinic, 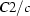

*a* = 26.628 (2) Å
*b* = 5.7109 (3) Å
*c* = 24.772 (1) Å
β = 100.396 (3)°
*V* = 3705.2 (4) Å^3^
*Z* = 8
Mo *K*α radiation
μ = 0.86 mm^−1^
*T* = 119 K
0.40 × 0.04 × 0.04 mm

#### Data collection

Bruker SMART APEX diffractometer
Absorption correction: multi-scan (*SADABS*; Sheldrick, 1996 ▶) *T*_min_ = 0.724, *T*_max_ = 0.966
11303 measured reflections
4189 independent reflections
2154 reflections with *I* > 2σ(*I*)
*R*_int_ = 0.174

#### Refinement

*R*[*F* ^2^ > 2σ(*F* ^2^)] = 0.059
*wR*(*F* ^2^) = 0.175
*S* = 0.97
4189 reflections
239 parameters
H-atom parameters constrained
Δρ_max_ = 1.40 e Å^−3^
Δρ_min_ = −1.20 e Å^−3^

### 

Data collection: *APEX2* (Bruker, 2008 ▶); cell refinement: *SAINT* (Bruker, 2008 ▶); data reduction: *SAINT*; program(s) used to solve structure: *SHELXS97* (Sheldrick, 2008 ▶); program(s) used to refine structure: *SHELXL97* (Sheldrick, 2008 ▶); molecular graphics: *X-SEED* (Barbour, 2001 ▶); software used to prepare material for publication: *publCIF* (Westrip, 2009 ▶).

## Supplementary Material

Crystal structure: contains datablocks global, I. DOI: 10.1107/S1600536809019928/xu2529sup1.cif

Structure factors: contains datablocks I. DOI: 10.1107/S1600536809019928/xu2529Isup2.hkl

Additional supplementary materials:  crystallographic information; 3D view; checkCIF report

## Figures and Tables

**Table 1:** Selected bond lengths (Å)

V1—N1	2.064 (4)
V1—N4	1.888 (4)
V1—O1	1.988 (4)
V1—O3	1.989 (3)
V1—O4	2.207 (4)
V1—O5	1.593 (4)

**Table 2:** Hydrogen-bond geometry (Å, °)

*D*—H⋯*A*	*D*—H	H⋯*A*	*D*⋯*A*	*D*—H⋯*A*
N3—H3⋯O2^i^	0.88	1.92	2.744 (5)	156
N4—H4⋯O1^i^	0.88	2.14	2.840 (5)	136

Symmetry code: (i) 
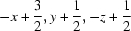
.

**Fig. 1.**
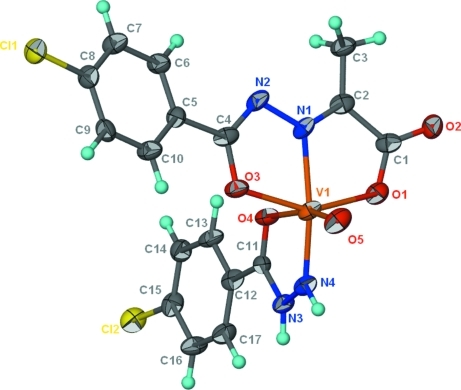
Thermal ellipsoid plot (Barbour, 2001) of VO(C7H6ClN2O)(C10H7ClN2O3) at the 70% probability level. Hydrogen atoms are drawn as spheres of arbitrary radius.

## supplementary crystallographic information

### Experimental

2-[*p*-Chlorobenzoylhydrazono]propionic acid was prepared from the condensation reaction of *p*-chlorobenzhydrazide and pyruvic acid. The compound (0.70 g, 3 mmol) and vanadyl sulfate (0.25 g, 1.5 mmol) in 20 ml of distilled water were heated for 5 h. Slow evaporation of the filtrate gave orange crystals.

### Refinement

Carbon-bound H-atoms were placed in calculated positions (C—H 0.95 to 0.98 Å) and were included in the refinement in the riding model approximation, with *U*(H) set to 1.2 to 1.5*U*(C). The nitrogen-bound H-atoms were similarly treated (N–H 0.88 Å).

All phenylene rings were refined was rigid hexagons of 1.39 Å sides.

The final difference Fourier map had a large peak/deep hole in the vicnity of the V1 atom.

### Crystal data

**Table:**

[V(C_7_H_6_ClN_2_O)(C_10_H_7_ClN_2_O_3_)O]	*F*(000) = 1920
*M**_r_* = 475.15	*D*_x_ = 1.704 Mg m^−^^3^
Monoclinic, *C*2/*c*	Mo *K*α radiation, λ = 0.71073 Å
Hall symbol: -C 2yc	Cell parameters from 957 reflections
*a* = 26.628 (2) Å	θ = 2.5–23.1°
*b* = 5.7109 (3) Å	µ = 0.86 mm^−^^1^
*c* = 24.772 (1) Å	*T* = 119 K
β = 100.396 (3)°	Prism, orange
*V* = 3705.2 (4) Å^3^	0.40 × 0.04 × 0.04 mm
*Z* = 8	

### Data collection

**Table:**

Bruker SMART APEX diffractometer	4189 independent reflections
Radiation source: fine-focus sealed tube	2154 reflections with *I* > 2σ(*I*)
graphite	*R*_int_ = 0.174
ω scans	θ_max_ = 27.5°, θ_min_ = 1.6°
Absorption correction: multi-scan (*SADABS*; Sheldrick, 1996)	*h* = −34→34
*T*_min_ = 0.724, *T*_max_ = 0.966	*k* = −7→7
11303 measured reflections	*l* = −32→30

### Refinement

**Table:**

Refinement on *F*^2^	Primary atom site location: structure-invariant direct methods
Least-squares matrix: full	Secondary atom site location: difference Fourier map
*R*[*F*^2^ > 2σ(*F*^2^)] = 0.059	Hydrogen site location: inferred from neighbouring sites
*wR*(*F*^2^) = 0.175	H-atom parameters constrained
*S* = 0.97	*w* = 1/[σ^2^(*F*_o_^2^) + (0.0714*P*)^2^] where *P* = (*F*_o_^2^ + 2*F*_c_^2^)/3
4189 reflections	(Δ/σ)_max_ = 0.001
239 parameters	Δρ_max_ = 1.40 e Å^−^^3^
0 restraints	Δρ_min_ = −1.20 e Å^−^^3^

### Fractional atomic coordinates and isotropic or equivalent isotropic displacement parameters (Å^2^)

**Table:**

	*x*	*y*	*z*	*U*_iso_*/*U*_eq_	
V1	0.66692 (3)	0.32434 (15)	0.27882 (4)	0.0216 (3)	
Cl1	0.44158 (5)	0.7483 (2)	0.46938 (6)	0.0289 (4)	
Cl2	0.84139 (5)	−0.6026 (2)	0.53131 (6)	0.0294 (4)	
O1	0.67515 (13)	0.1122 (6)	0.21738 (17)	0.0236 (9)	
O2	0.63441 (13)	−0.1245 (6)	0.15164 (17)	0.0281 (9)	
O3	0.62899 (12)	0.4195 (6)	0.33748 (16)	0.0237 (9)	
O4	0.69433 (12)	0.0349 (6)	0.33560 (16)	0.0222 (9)	
O5	0.65919 (13)	0.5607 (6)	0.24415 (18)	0.0296 (10)	
N1	0.60013 (15)	0.1326 (7)	0.2650 (2)	0.0203 (10)	
N2	0.56469 (15)	0.1678 (7)	0.2986 (2)	0.0236 (11)	
N3	0.76603 (15)	0.2341 (7)	0.34041 (19)	0.0206 (10)	
H3	0.7990	0.2555	0.3516	0.025*	
N4	0.73671 (15)	0.3820 (7)	0.3060 (2)	0.0215 (10)	
H4	0.7506	0.5106	0.2958	0.026*	
C1	0.63701 (18)	−0.0159 (9)	0.1950 (3)	0.0228 (12)	
C2	0.59319 (18)	−0.0213 (9)	0.2266 (2)	0.0225 (12)	
C3	0.55037 (18)	−0.1879 (9)	0.2136 (3)	0.0280 (14)	
H3A	0.5521	−0.3016	0.2435	0.042*	
H3B	0.5526	−0.2702	0.1794	0.042*	
H3C	0.5180	−0.1025	0.2092	0.042*	
C4	0.58356 (18)	0.3289 (8)	0.3341 (3)	0.0221 (12)	
C5	0.55047 (11)	0.4234 (5)	0.37140 (15)	0.0196 (12)	
C6	0.50488 (12)	0.3112 (5)	0.37506 (15)	0.0239 (13)	
H6	0.4965	0.1671	0.3565	0.029*	
C7	0.47159 (10)	0.4100 (5)	0.40592 (17)	0.0249 (13)	
H7A	0.4404	0.3333	0.4084	0.030*	
C8	0.48390 (11)	0.6209 (5)	0.43310 (15)	0.0227 (12)	
C9	0.52949 (12)	0.7331 (5)	0.42944 (15)	0.0239 (12)	
H9	0.5379	0.8772	0.4480	0.029*	
C10	0.56278 (10)	0.6343 (5)	0.39859 (16)	0.0254 (13)	
H10	0.5939	0.7110	0.3961	0.031*	
C11	0.74052 (18)	0.0506 (8)	0.3562 (2)	0.0186 (12)	
C12	0.76740 (11)	−0.1137 (5)	0.39758 (13)	0.0222 (13)	
C13	0.73990 (8)	−0.3013 (5)	0.41306 (14)	0.0245 (13)	
H13	0.7054	−0.3249	0.3958	0.029*	
C14	0.76286 (11)	−0.4543 (5)	0.45377 (15)	0.0241 (12)	
H14	0.7441	−0.5825	0.4644	0.029*	
C15	0.81333 (11)	−0.4198 (5)	0.47899 (13)	0.0251 (13)	
C16	0.84083 (9)	−0.2323 (6)	0.46350 (14)	0.0273 (13)	
H16	0.8753	−0.2087	0.4807	0.033*	
C17	0.81787 (10)	−0.0792 (5)	0.42280 (15)	0.0251 (13)	
H17	0.8367	0.0489	0.4122	0.030*	

### Atomic displacement parameters (Å^2^)

**Table:**

	*U*^11^	*U*^22^	*U*^33^	*U*^12^	*U*^13^	*U*^23^
V1	0.0156 (4)	0.0218 (5)	0.0292 (7)	0.0012 (4)	0.0087 (4)	0.0014 (4)
Cl1	0.0244 (6)	0.0351 (8)	0.0291 (9)	0.0057 (5)	0.0097 (6)	−0.0082 (6)
Cl2	0.0273 (7)	0.0282 (7)	0.0332 (10)	0.0029 (6)	0.0066 (6)	0.0046 (7)
O1	0.0189 (17)	0.0246 (19)	0.028 (2)	0.0046 (15)	0.0074 (17)	0.0015 (17)
O2	0.0217 (18)	0.040 (2)	0.024 (3)	0.0046 (17)	0.0079 (17)	−0.004 (2)
O3	0.0151 (16)	0.0256 (19)	0.031 (3)	−0.0022 (14)	0.0067 (17)	−0.0060 (18)
O4	0.0150 (16)	0.0225 (18)	0.029 (3)	−0.0032 (14)	0.0048 (17)	−0.0022 (17)
O5	0.0269 (19)	0.025 (2)	0.039 (3)	0.0037 (16)	0.013 (2)	0.0060 (19)
N1	0.018 (2)	0.019 (2)	0.027 (3)	0.0043 (17)	0.011 (2)	0.003 (2)
N2	0.018 (2)	0.024 (2)	0.032 (3)	0.0009 (18)	0.014 (2)	−0.004 (2)
N3	0.0137 (19)	0.025 (2)	0.023 (3)	0.0018 (17)	0.0037 (18)	0.005 (2)
N4	0.020 (2)	0.018 (2)	0.028 (3)	−0.0018 (17)	0.009 (2)	0.000 (2)
C1	0.016 (2)	0.020 (3)	0.033 (4)	0.003 (2)	0.004 (2)	−0.001 (3)
C2	0.016 (2)	0.021 (3)	0.030 (4)	0.004 (2)	0.004 (2)	0.003 (3)
C3	0.018 (2)	0.032 (3)	0.036 (4)	−0.004 (2)	0.010 (3)	−0.007 (3)
C4	0.018 (2)	0.017 (2)	0.032 (4)	0.000 (2)	0.005 (2)	0.004 (3)
C5	0.019 (2)	0.020 (3)	0.021 (3)	0.002 (2)	0.006 (2)	−0.003 (2)
C6	0.022 (2)	0.021 (3)	0.029 (4)	−0.003 (2)	0.006 (2)	−0.007 (3)
C7	0.021 (3)	0.021 (3)	0.034 (4)	0.000 (2)	0.009 (3)	−0.001 (3)
C8	0.022 (2)	0.023 (3)	0.023 (3)	0.005 (2)	0.006 (2)	−0.004 (2)
C9	0.026 (3)	0.026 (3)	0.019 (3)	0.000 (2)	0.003 (2)	−0.005 (2)
C10	0.017 (2)	0.028 (3)	0.033 (4)	−0.001 (2)	0.006 (2)	−0.003 (3)
C11	0.017 (2)	0.022 (3)	0.017 (3)	−0.003 (2)	0.005 (2)	−0.004 (2)
C12	0.019 (2)	0.018 (3)	0.031 (4)	0.002 (2)	0.011 (2)	−0.008 (2)
C13	0.016 (2)	0.024 (3)	0.036 (4)	−0.003 (2)	0.011 (2)	−0.006 (3)
C14	0.026 (3)	0.024 (3)	0.023 (3)	−0.002 (2)	0.006 (2)	0.000 (3)
C15	0.023 (3)	0.027 (3)	0.027 (4)	0.007 (2)	0.009 (3)	0.002 (3)
C16	0.014 (2)	0.032 (3)	0.035 (4)	−0.001 (2)	0.003 (2)	0.003 (3)
C17	0.023 (3)	0.024 (3)	0.029 (4)	−0.003 (2)	0.007 (3)	0.005 (3)

### Geometric parameters (Å, °)

**Table:**

V1—N1	2.064 (4)	C3—H3C	0.9800
V1—N4	1.888 (4)	C4—C5	1.489 (5)
V1—O1	1.988 (4)	C5—C6	1.3900
V1—O3	1.989 (3)	C5—C10	1.3900
V1—O4	2.207 (4)	C6—C7	1.3900
V1—O5	1.593 (4)	C6—H6	0.9500
Cl1—C8	1.724 (2)	C7—C8	1.3900
Cl2—C15	1.726 (3)	C7—H7A	0.9500
O1—C1	1.293 (6)	C8—C9	1.3900
O2—C1	1.231 (6)	C9—C10	1.3900
O3—C4	1.305 (5)	C9—H9	0.9500
O4—C11	1.247 (6)	C10—H10	0.9500
N1—C2	1.284 (7)	C11—C12	1.476 (6)
N1—N2	1.381 (5)	C12—C13	1.3900
N2—C4	1.308 (7)	C12—C17	1.3900
N3—N4	1.344 (6)	C13—C14	1.3900
N3—C11	1.345 (6)	C13—H13	0.9500
N3—H3	0.8800	C14—C15	1.3900
N4—H4	0.8800	C14—H14	0.9500
C1—C2	1.518 (7)	C15—C16	1.3900
C2—C3	1.475 (7)	C16—C17	1.3900
C3—H3A	0.9800	C16—H16	0.9500
C3—H3B	0.9800	C17—H17	0.9500
			
O5—V1—N4	93.89 (19)	O3—C4—C5	117.5 (4)
O5—V1—O1	97.17 (18)	N2—C4—C5	118.3 (4)
N4—V1—O1	98.17 (16)	C6—C5—C10	120.0
O5—V1—O3	97.41 (17)	C6—C5—C4	119.7 (3)
N4—V1—O3	106.77 (17)	C10—C5—C4	120.1 (3)
O1—V1—O3	150.05 (14)	C5—C6—C7	120.0
O5—V1—N1	109.67 (19)	C5—C6—H6	120.0
N4—V1—N1	156.21 (18)	C7—C6—H6	120.0
O1—V1—N1	76.15 (15)	C8—C7—C6	120.0
O3—V1—N1	74.34 (15)	C8—C7—H7A	120.0
O5—V1—O4	166.80 (18)	C6—C7—H7A	120.0
N4—V1—O4	73.27 (16)	C9—C8—C7	120.0
O1—V1—O4	87.83 (14)	C9—C8—Cl1	120.02 (18)
O3—V1—O4	83.82 (14)	C7—C8—Cl1	119.94 (18)
N1—V1—O4	83.36 (15)	C10—C9—C8	120.0
C1—O1—V1	119.1 (3)	C10—C9—H9	120.0
C4—O3—V1	115.6 (3)	C8—C9—H9	120.0
C11—O4—V1	113.3 (3)	C9—C10—C5	120.0
C2—N1—N2	121.6 (4)	C9—C10—H10	120.0
C2—N1—V1	119.4 (3)	C5—C10—H10	120.0
N2—N1—V1	119.0 (3)	O4—C11—N3	116.7 (5)
C4—N2—N1	106.6 (4)	O4—C11—C12	123.9 (4)
N4—N3—C11	114.1 (4)	N3—C11—C12	119.4 (4)
N4—N3—H3	123.0	C13—C12—C17	120.0
C11—N3—H3	123.0	C13—C12—C11	117.9 (3)
N3—N4—V1	122.6 (3)	C17—C12—C11	122.0 (3)
N3—N4—H4	118.7	C12—C13—C14	120.0
V1—N4—H4	118.7	C12—C13—H13	120.0
O2—C1—O1	125.1 (5)	C14—C13—H13	120.0
O2—C1—C2	120.8 (5)	C13—C14—C15	120.0
O1—C1—C2	114.1 (5)	C13—C14—H14	120.0
N1—C2—C3	127.3 (5)	C15—C14—H14	120.0
N1—C2—C1	110.3 (4)	C16—C15—C14	120.0
C3—C2—C1	122.4 (5)	C16—C15—Cl2	119.67 (19)
C2—C3—H3A	109.5	C14—C15—Cl2	120.29 (19)
C2—C3—H3B	109.5	C15—C16—C17	120.0
H3A—C3—H3B	109.5	C15—C16—H16	120.0
C2—C3—H3C	109.5	C17—C16—H16	120.0
H3A—C3—H3C	109.5	C16—C17—C12	120.0
H3B—C3—H3C	109.5	C16—C17—H17	120.0
O3—C4—N2	124.2 (5)	C12—C17—H17	120.0
			
O5—V1—O1—C1	−101.5 (4)	O1—C1—C2—N1	9.8 (6)
N4—V1—O1—C1	163.5 (4)	O2—C1—C2—C3	12.0 (8)
O3—V1—O1—C1	17.1 (6)	O1—C1—C2—C3	−169.0 (5)
N1—V1—O1—C1	7.1 (4)	V1—O3—C4—N2	6.8 (7)
O4—V1—O1—C1	90.8 (4)	V1—O3—C4—C5	−170.4 (3)
O5—V1—O3—C4	104.1 (4)	N1—N2—C4—O3	−4.5 (7)
N4—V1—O3—C4	−159.6 (4)	N1—N2—C4—C5	172.6 (4)
O1—V1—O3—C4	−14.5 (5)	O3—C4—C5—C6	−170.8 (4)
N1—V1—O3—C4	−4.3 (4)	N2—C4—C5—C6	11.9 (6)
O4—V1—O3—C4	−89.1 (4)	O3—C4—C5—C10	15.1 (6)
O5—V1—O4—C11	−14.2 (9)	N2—C4—C5—C10	−162.2 (4)
N4—V1—O4—C11	−0.7 (3)	C10—C5—C6—C7	0.0
O1—V1—O4—C11	98.5 (3)	C4—C5—C6—C7	−174.2 (4)
O3—V1—O4—C11	−110.3 (3)	C5—C6—C7—C8	0.0
N1—V1—O4—C11	174.8 (4)	C6—C7—C8—C9	0.0
O5—V1—N1—C2	91.9 (4)	C6—C7—C8—Cl1	177.7 (3)
N4—V1—N1—C2	−79.5 (6)	C7—C8—C9—C10	0.0
O1—V1—N1—C2	−0.8 (4)	Cl1—C8—C9—C10	−177.7 (3)
O3—V1—N1—C2	−175.6 (4)	C8—C9—C10—C5	0.0
O4—V1—N1—C2	−90.2 (4)	C6—C5—C10—C9	0.0
O5—V1—N1—N2	−90.2 (4)	C4—C5—C10—C9	174.1 (4)
N4—V1—N1—N2	98.4 (5)	V1—O4—C11—N3	−1.1 (6)
O1—V1—N1—N2	177.0 (4)	V1—O4—C11—C12	176.7 (3)
O3—V1—N1—N2	2.2 (3)	N4—N3—C11—O4	3.1 (7)
O4—V1—N1—N2	87.6 (4)	N4—N3—C11—C12	−174.9 (4)
C2—N1—N2—C4	178.1 (5)	O4—C11—C12—C13	2.1 (6)
V1—N1—N2—C4	0.3 (5)	N3—C11—C12—C13	179.9 (3)
C11—N3—N4—V1	−4.0 (6)	O4—C11—C12—C17	−174.7 (4)
O5—V1—N4—N3	179.5 (4)	N3—C11—C12—C17	3.1 (5)
O1—V1—N4—N3	−82.7 (4)	C17—C12—C13—C14	0.0
O3—V1—N4—N3	80.5 (4)	C11—C12—C13—C14	−176.9 (3)
N1—V1—N4—N3	−8.6 (7)	C12—C13—C14—C15	0.0
O4—V1—N4—N3	2.5 (4)	C13—C14—C15—C16	0.0
V1—O1—C1—O2	167.7 (4)	C13—C14—C15—Cl2	177.6 (3)
V1—O1—C1—C2	−11.3 (6)	C14—C15—C16—C17	0.0
N2—N1—C2—C3	−3.4 (8)	Cl2—C15—C16—C17	−177.6 (3)
V1—N1—C2—C3	174.5 (4)	C15—C16—C17—C12	0.0
N2—N1—C2—C1	177.9 (4)	C13—C12—C17—C16	0.0
V1—N1—C2—C1	−4.3 (6)	C11—C12—C17—C16	176.8 (3)
O2—C1—C2—N1	−169.2 (5)		

### Hydrogen-bond geometry (Å, °)

**Table:**

*D*—H···*A*	*D*—H	H···*A*	*D*···*A*	*D*—H···*A*
N3—H3···O2^i^	0.88	1.92	2.744 (5)	156
N4—H4···O1^i^	0.88	2.14	2.840 (5)	136

Symmetry codes: (i) −*x*+3/2, *y*+1/2, −*z*+1/2.
